# DCNAS-Net: deformation convolution and neural architecture search detection network for bone marrow oedema

**DOI:** 10.1186/s12880-023-01003-8

**Published:** 2023-03-28

**Authors:** Chengyu Song, Shan Zhu, Yanyan Liu, Wei Zhang, Zhi Wang, Wangxiao Li, Zhenye Sun, Peng Zhao, Shengzhang Tian

**Affiliations:** 1 Tianjin University, Tianjin, China; 2 Tianjin Hospital, Tianjin University, Tianjin, China; 3 Nankai University, Tianjin, China

**Keywords:** Lumbago MRI, Bone marrow oedema, Neural networks, Target detection

## Abstract

**Background:**

Lumbago is a global disease that affects more than 500 million people worldwide. Bone marrow oedema is one of the main causes of the condition and clinical diagnosis is mainly made by radiologists manually reviewing MRI images to determine whether oedema is present. However, the number of patients with Lumbago has risen dramatically in recent years, which has brought a huge workload to radiologists. In order to improve the efficiency of diagnosis, this paper is devoted to developing and evaluating a neural network for detecting bone marrow edema in MRI images.

**Related work:**

Inspired by the development of deep learning and image processing techniques, we design a deep learning detection algorithm specifically for the detection of bone marrow oedema from lumbar MRI images. We introduce deformable convolution, feature pyramid networks and neural architecture search modules, and redesign the existing neural networks. We explain in detail the construction of the network and illustrate the setting of the network hyperparameters.

**Results and discussion:**

The detection accuracy of our algorithm is excellent. And its accuracy of detecting bone marrow oedema reached up to 90.6$\%$, an improvement of 5.7$\%$ compared to the original. The recall of our neural network is 95.1$\%$, and the F1-measure also reaches 92.8$\%$. And our algorithm is fast in detecting it, taking only 0.144 s per image.

**Conclusion:**

Extensive experiments have demonstrated that deformable convolution and aggregated feature pyramid structures are conducive for the detection of bone marrow oedema. Our algorithm has better detection accuracy and good detection speed compared to other algorithms.

## Introduction and background

Lumbago is one of the major global health problems, with a prevalence of 11–84$\%$ [1]. According to the Global Burden of Disease, lumbago troubles over 500 million people in 2015, 17.3$\%$ higher than that in 2005, and it is one of the leading causes of disability in most countries [2]. The clinical assessment of lumbago involves taking a correct history and performing a thorough physical examination and imaging of the low back [3]. There are many causes of lumbago, of which bone marrow oedema is the most common one. Bone marrow oedema in the lumbar region is mainly examined clinically by MRI. Typically, patients have MRI images of the lumbar region taken with medical equipment and these MRI images are described in a radiology report by a radiologist who are responsible for evaluating and tracking the lesion site quantitatively [4]. With the dramatic increase in the number of patients suffering from bone marrow oedema in recent years, the corresponding number of MRI images has also increased dramatically. The enormous workload of reviewing the images has put great pressure on the doctors concerned, making them more prone to misdiagnosis and missed diagnoses, which has caused great disturbance to doctors and patients’ recovery. As a result, new techniques are urgently needed to be applied to this field.

In this paper, we design a new neural network to detect bone marrow edema. Different from the general classification network, our network can not only accurately determine whether the MRI image is abnormal, but also accurately give the location of bone marrow edema. The paper is organized as follows. First, we discuss the research and application of deep learning in MRI image processing at present. Then, aiming at the problem of bone marrow edema detection, we introduce deformable convolution module and multilevel feature pyramid structure to improve the detection effect of neural network, and introduce the latest neural structure search module to streamline the network. Then we proved the effectiveness of our improvement through a large number of experiments. Finally, we summarize and look forward to the research work of this paper.

## Related work

In recent years, with the progress of image processing technology, researchers have developed CAD (Computer Aided Detection) systems, which are able to be partly automated in diagnostic process. At the same time, deep learning shines in the field of image processing. The medical image detection based on deep learning has also made great progress. Different from the traditional methods, the deep learning method is based on the characteristics of images and targets to learn features, which can effectively identify images and targets with different styles. At the same time, the deep learning algorithm is easier to design and deploy. Therefore, researchers actively apply the idea of deep learning to the field of medical images, including a large number of MRI image processing and analysis. For example, Liao et al. [5] proposed a neural network model for automatic detection of lung nodules by improving the U-net network. They added a 3D convolutional module to the U-net network to increase the accuracy of detecting lung nodules in MRI images. Dezso Ribli et al. [6] proposed a CAD system based on the Faster-R-CNN, which improved the network by employing a reactive propagation mechanism with weight decay and a stochastic gradient descent mechanism, and it effectively improved the detection accuracy of malignant breast lesions. Huang Tao et al. [7] designed a neural network specifically for grading the severity of pleural effusion. It effectively improved the accuracy of pleural effusion grading by designing three data processing methods and two loss functions. In the field of bone marrow disease detection, Klontzas et al. [8] designed a classification model based on CNN ensemble. They combined three kinds of CNN, VGG-16, Inception-V3 and Inception-ResNet-V2, into a CNN ensemble. In their experiment, the AUC(Area Under Curve) of the model for detecting bone marrow diseases increased from 93.68 to 95.97$\%$. And the detection effect of this CNN ensemble has reached the same or higher performance of a radiologist. Lee et al. [9] develop a method for detecting bone marrow edema by MRI of the sacroiliac joints and a deep-learning network. It is found that the classification network based on ResNet18 has the best performance, which shows that the detection accuracy of bone marrow edema can reach 94.59$\%$. Their research suggests that analysis based on deep learning can be an effective supplementary means for clinicians to diagnose bone marrow edema.

**Fig. 1:**
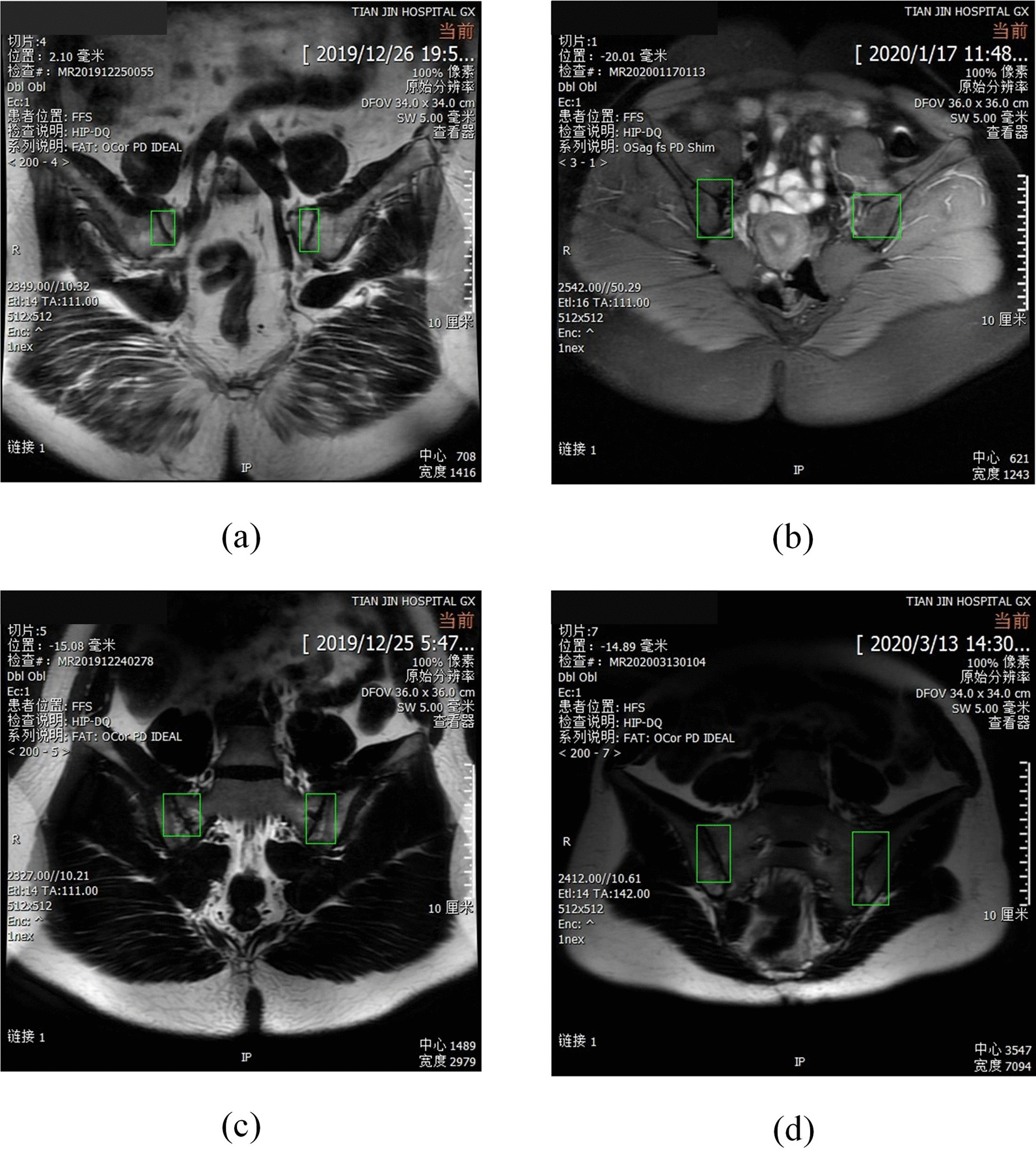
Different styles of MRI images and different shapes of bone marrow edema. **a** Easily distinguishable bone marrow oedema; **b** Indistinguishable bone marrow oedema; **c** Indistinguishable bone marrow oedema; **d** Indistinguishable bone marrow oedema

However, in the field of medical image processing, most of the work combined with deep learning focuses on image classification. Although the classification task can quickly identify the abnormal images, it can’t accurately point out the abnormal positions on the images. For MRI images of lumbar region, there are many organs and tissues, which are similar to the features of bone marrow edema. Coupled with the diversity in the size and shape of bone marrow oedema, current network models perform poor effectiveness in detecting bone marrow oedema in MRI of the lumbar region. As shown in Fig. 1, bone marrow edema varies greatly on different MRI images. We can easily find out the bone marrow edema in Fig. 1a, while Fig. 1b–d needs more careful observation to find it. Moreover, the background elements in MRI images are very different due to the different slices, which will further reduce the accuracy of bone marrow edema detection. In general, there are three main problems with the detection of bone marrow oedema:

- Variable shape and size of bone marrow edema on MRI of the lumbar region. Conventional convolution networks are not effective in extracting irregular targets, especially bone marrow oedema.
- Lumbar MRI images contain a large amount of organ tissue, and bone marrow oedema has a high degree of similarity to these. In such images, conventional feature extraction modules are unable to accurately extract the features of weak targets such as bone marrow oedema.
- MRI images taken by different models of equipment can be significantly different. Conventional algorithms are generally only effective for fixed style images and are less effective in processing MRI images from different devices.

In order to solve these problems, we design a new neural network to detect bone marrow oedema in MRI images. Our work is inspired by the idea of the deformable convolution of DCN [10] networks. We replace the regular convolution and pooling modules of the feature extraction layer with deformable convolution and deformable RoI (Region of Interest) pooling modules, which can improve the detection ability of bone marrow edema targets with different shapes. In addition, on the basis of the FPN (Feature Pyramid Networks) optimization ideas in networks such as FPN [11], PA-Net [12], Libra R-CNN [13], SEPC-Neck [14], BIFPN [15] and OPANAS [16], our research introduces one single path feature aggregation module based on NAS (Neural Architecture Search) [17, 18]. The module automatically searches for the optimal feature extraction module by means of 4 unique feature extraction paths. It improves the ability to extract smaller bone marrow oedema in MRI images. In addition to this, our algorithm inserts 2 special parameter-free information paths, which can reduce the redundancy of the algorithm and the phenomenon of overfitting. Because of the combination of the deformable convolution module and the NAS module, our algorithm is called DCNAS-Net (Deformable Convolution and Neural Architecture Search Networks).

## Methods

### Structure of DCNAS-Net

**Fig. 2:**
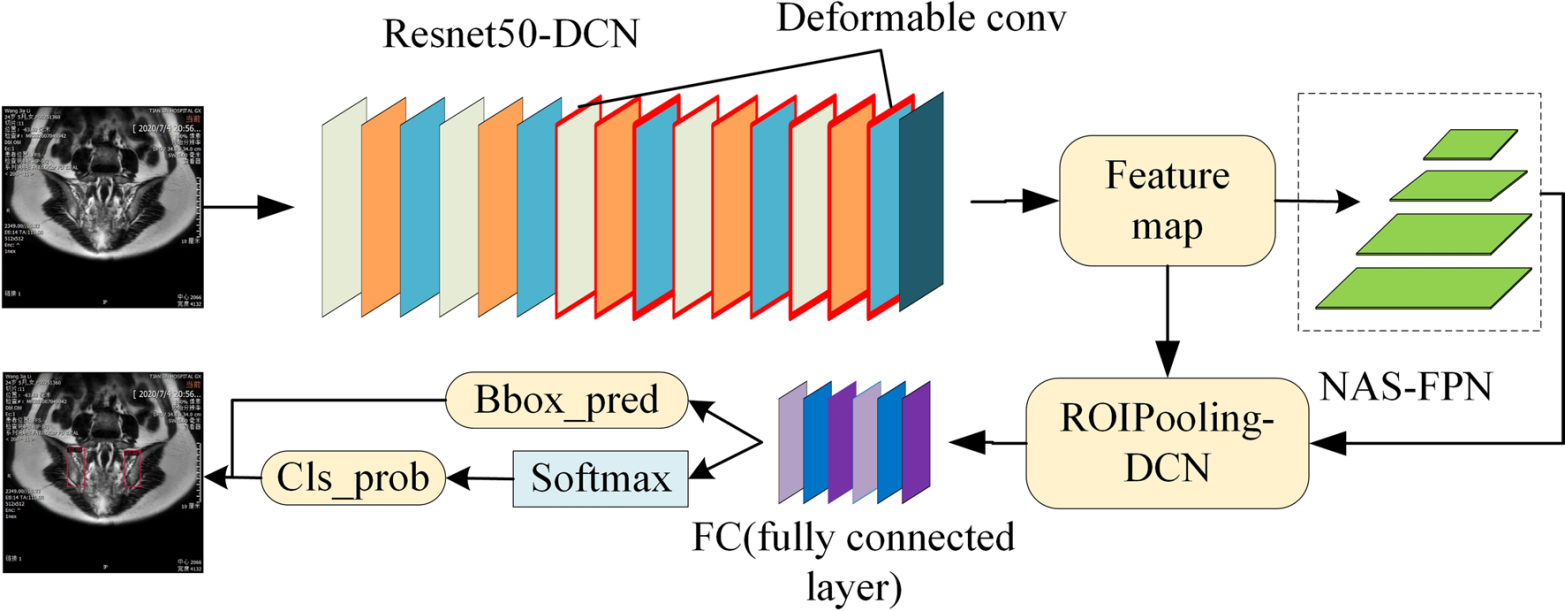
Structure of the DCNAS-Net

The structure of DCNAS-Net is shown in Fig. 2. DCNAS-Net is structured with ResNet-50 as backbone, with the addition of the deformable convolution module in conv3-conv5. Then it is followed by the NAS-FPN module. It constructs the automatic search space by means of 4 unique information paths and 2 parameter-free information paths. 6 paths form different connections between the backbone network and the detection head. It will form a complementary and efficient aggregation module. The output of the NAS-FPN is superimposed on the feature matrix of the backbone. Then there is the deformable RoI pool layer. Finally, a 1024-dimensional fully connected layer is accessed. The inference information is then returned to the original graph by prediction frames and predictive classification.

### Deformable convolution and deformable RoI pooling layers

**Fig. 3:**
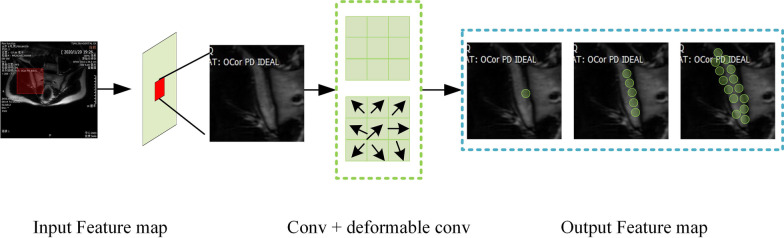
Mechanism of deformable convolution. The cropped image is realized by python script, and it contains the bone marrow edema area

One of the factors why bone marrow edema is difficult to be detected in MRI images is its variable size and shape. The conventional convolution module is limited to a fixed ensemble structure and its convolution unit samples the input feature map at a fixed location. In addition, the pooling layer reduces the spatial resolution by a fixed ratio. Obviously, it lacks the internal mechanism for geometric transformations. Therefore, it performs poorly when detecting variable shaped targets, especially in cases such as bone marrow oedema. In general, expanding the data samples could solve this problem to some extent. However, due to the specific nature of medical data, it is impossible to collect a large amount of data. To address this problem we add the deformable convolution and deformable RoI pooling layer module to the backbone network. The two modules can improve the ability of the backbone network to extract irregularly shaped features. The working mechanism of the deformable convolution is shown as Fig. 3. We add a 2D offset to the standard convolution module, which allows sampling grids to be freely deformed and enables the neural network to adapt to the different shapes of the bone marrow oedema. The density of deformable convolution and its deformation mode depend on the input feature layer. Our research incorporates an additional layer of deformable convolution so that the offset of the features can be learned from the upper feature layer. The green dotted box in Fig. 3 shows the offset (black arrow) of the deformable convolution based on the standard convolution. And the blue dotted box in Fig. 3 indicates the feature extraction process in the upsampling process. In the training process, the feature extraction layer is activated by a single activation unit located in the target area. The offset of features will be determined according to the location and shape of bone marrow edema. In Fig. 3, the feature offset will be more inclined to the upper left and lower right in the initial position of the activation unit. After several rounds of training, the output feature layer will be able to better learn the features of the bone marrow edema area.

And then it comes to the deformable RoI pooling layer [19, 20]. This module adds an offset after the RoI pooling layer, and the structure is useful for improving the accuracy of local positioning of targets. Both of these modules are lightweight modules. They can be inserted into a regular network by adding a small number of parameters and computational effort. The construction of the deformable convolution module and the deformable RoI pooling layer are described below. A conventional convolution is built in two steps:

- Sampling the input feature mapping *x* using a regular grid *R*;
- Weighting and summing the sampled values.

Defining *R* as the size of the perceptual field, and taking a $3 \times 3$ convolution as an example, with a convolution hole size of 1, we have

1 $$\begin{aligned} \begin{matrix} {R = \left\{ {\left( {- 1, - 1} \right) ,\left( {- 1,0} \right) ,\ldots ,(0,1),(1,1)} \right\} } \end{matrix} \end{aligned}$$

For $p_0$, each position of the input feature map *y*, we have

2 $$\begin{aligned} y\left( p_{0} \right) = {\sum \limits _{p_{0} \in {\mathcal {R}}}{w\left( p_{0} \right) \times x\left( {p_{0} + p_{n}} \right) }} \end{aligned}$$

Where $p_n$ enumerates all the positions in *R*. In the deformable convolution, an offset $\mathrm {\Delta }p_{n}\left( \big | n = 1,2,\ldots ,N \right)$ is added to the regular sampling grid *R*, and Eq. (2) becomes

3 $$\begin{aligned} y\left( p_{0} \right) = {\sum \limits _{p_{0} \in {\mathcal {R}}}{w\left( p_{0} \right) \times x\left( {p_{0} + p_{n} + \mathrm {\Delta }p_{n}} \right) }} \end{aligned}$$

The sampling grid samples at offset position $p_{n} + \mathrm {\Delta }p_{n}$. In most cases, the offset $\mathrm {\Delta }p_{n}$ is generally not an integer. Then Eq. (3) can be implemented by bilinear difference as follows

4 $$\begin{aligned} x(p) = {\sum \limits _{q}{G\left( {q,p} \right) \times }}x(q) \end{aligned}$$

Where *p* denotes any position in *R*, *q* is an enumeration of all integer positions in *x*. *G*(*q*, *p*) is a 2D bilinear interpolation kernel. It can be decomposed as the product of two 1D kernels as

5 $$\begin{aligned} G\left( {q,p} \right) = g\left( q_{x},p_{x} \right) \times g\left( q_{y},p_{y} \right) \end{aligned}$$

Where $g\left( {a,b} \right) = max\left( 0,1 - \big | a - b \big | \right)$, it follows that *G*(*q*, *p*) is not equal to 0 for only a few *q*, so it does not consume many computational resources.

### Deformable RoI pooling layer

The role of the RoI pooling layer [21] is to unify the feature matrices corresponding to RoI of different sizes into a fixed size bin for output. For a conventional RoI pooling layer, assuming that the input feature matrix is *x*, its size is $w \times h$, $p_0$ is its coordinate, and *y*(*i*, *j*) denotes the bin at (*i*, *j*), we have

6 $$\begin{aligned} y\left( {i,j} \right) = {\sum \limits _{p \in bin(i,j)}{x\left( {p_{0} + p} \right) /}}n_{ij} \end{aligned}$$

Where $n_{ij}$ denotes the number of pixels in the bin at position (*i*, *j*). For the deformable pooling layer, we have

7 $$\begin{aligned} y\left( {i,j} \right) = {\sum \limits _{p \in bin(i,j)}{x\left( {p_{0} + p + \mathrm {\Delta }p_{ij}} \right) /}}n_{ij} \end{aligned}$$

Typically, $\mathrm {\Delta }p$ is also not an integer, so Eq. (7) can still be implemented with the bilinear difference of Eq. (4). In fact, the offsets in the deformable convolution and deformable RoI pooling layers can also be considered as parts of the network. It is obtained by adding an offset to the standard convolution, which in turn can also be learned end-to-end by back-passing of gradients. The size and position of the convolution kernels can be dynamically adjusted to the current target shape after adding the offset. The intuitive effect is that the sampling points of the convolutional kernels at different locations will change adaptively according to the shape of the bone marrow oedema, which can improve the detection accuracy.

### NAS based feature aggregation module

**Fig. 4:**
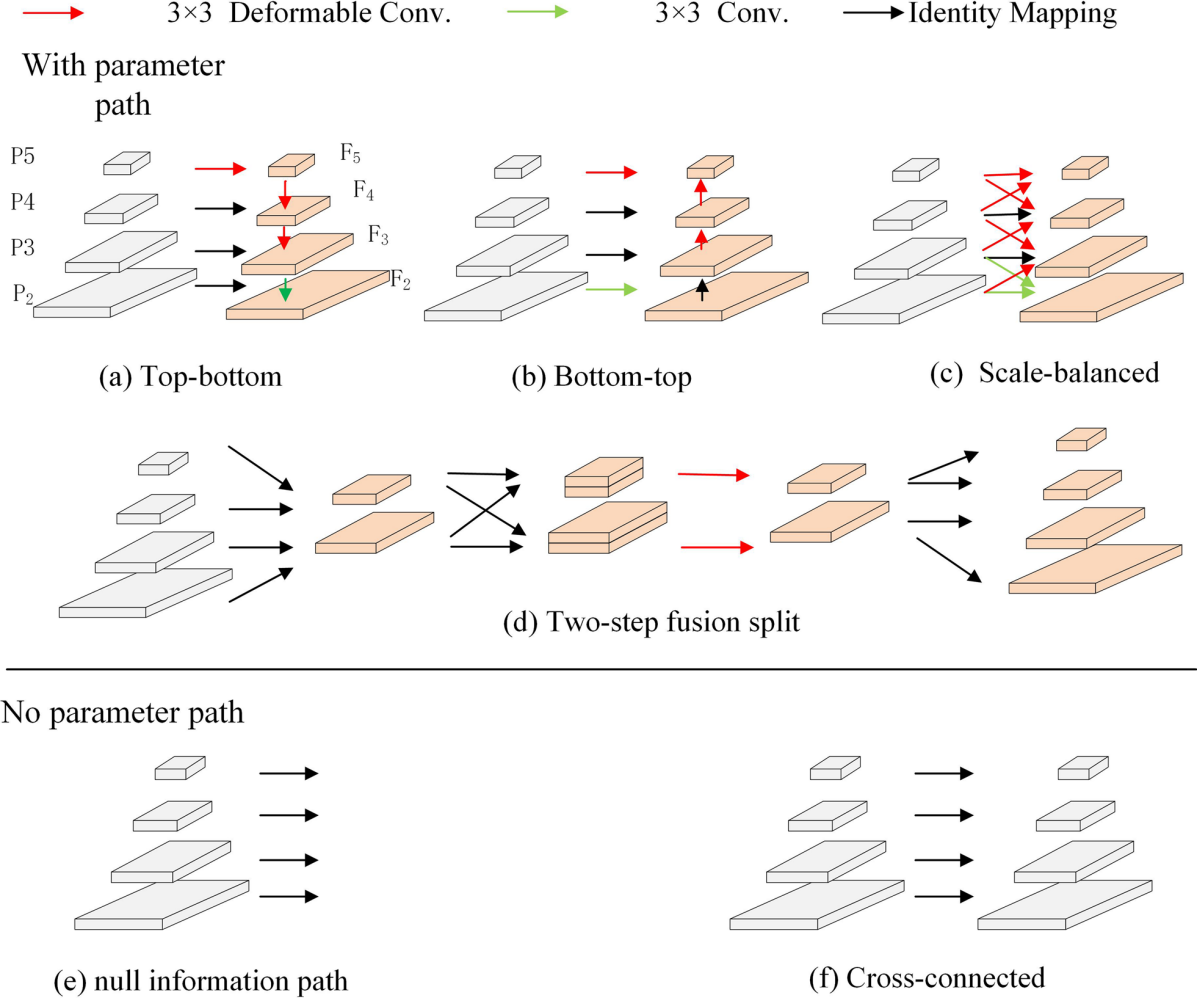
4 message paths with parameters and 2 message paths without parameters. **a** Top-bootom information path; **b** Bootom-top information path; **c** Scale-balanced information path; **d** Two-step fusion split information path; **e** Null information path; **f** Cross-connected information path

MRI images of the lumbar region are generally dominated by a grey background. Bone marrow oedema and some organ tissues,especially fatty tissues, are bright colors, which can make it difficult to identify bone marrow oedema. Even experienced radiologists take a few minutes to distinguish edema from other organs or tissues. For conventional neural networks, as the convolution layer deepens, the features extracted by the shallow convolution take up very little weight in the subsequent feature layers. By the time the convolution reaches the final layer, it is no longer possible to read the semantic information in the shallow background. That makes it difficult for conventional networks to detect small targets. Several studies in recent years have confirmed that the introduction of new information paths in the FPN structure can build more effective detection heads. Among them, PAFPN introduces bottom-up information paths on top of FPN; Libra R-CNN uses Non-Local path modules and builds multi-level FPN structures with U-shaped structures to construct more effective detection modules; SPEC stacks four scale-equilibrium modules behind a classical FPN to enhance multi-scale correlation. Meanwhile, research on NAS being used to automatically search for detectors in FPN architectures is also underway. NAS-FCOS and Spine-Net [22] have achieved excellent results after incorporating NAS. These prove that NAS architectures are effective. Inspired by the above studies our algorithm introduces four unique information paths and two non-reference paths to construct a multi-level FPN structure. The optimal FPN combination module is automatically searched by the latest efficient NAS module. This effectively improves the accuracy of bone marrow oedema detection.

#### Methods for constructing 6 information paths

- Top-down information path The top-down information path is similar to the classical FPN. Its structure is shown in Fig. 4a. We denote the output of the feature pyramid as $F_{2}^{t},F_{3}^{t},F_{4}^{t},F_{5}^{t}$, and then construct them in a top-down sequence. Each feature mapping $F_{i}^{t}$ is constructed by summing the input pyramid feature mapping $P_{i}$ at the same level and the output features $F_{i + 1}^{t}$ at a higher level, so that we have
  8 $$\begin{aligned} F_{i}^{t} = W_{i}^{t}\bigotimes \left( U\left( F_{i + 1}^{t} \right) + P_{i} \right) \end{aligned}$$
  Where $U( \cdot )$ denotes the upsampling function and $W_{i}^{t}(i = 2,3,4,5)$ are $3 \times 3$ convolution module. However, note that for the higher-level features $(i = 3,4,5)$, $W_{i}^{t}$ is a conformable convolution module, which can be better adapted to different pyramidal accesses, while $W_{2}^{t}$ is a regular convolution module.
- Bottom-up information path The structure of the bottom-up information path is shown as Fig. 4b, which is constructed in a similar way to the previous path. The outputs of the feature pyramid $\left( F_{2}^{b},F_{3}^{b},F_{4}^{b},F_{5}^{b} \right)$ are constructed sequentially in a bottom-up manner. We have
  9 $$\begin{aligned} F_{i}^{b} = W_{i}^{b}\bigotimes \left( D\left( F_{i - 1}^{b} \right) + P_{i} \right) \end{aligned}$$
  Where $D( \cdot )$ denotes the downsampling function. The convolution module of $W_{i}^{b}$ is the same as the top-down information path.
- Scale-balanced information path The scale-balanced information path is driven by SEPC, which superimposes a pyramidal convolution after the classical FPN structure to obtain the correlation among scales. Its structure is shown as Fig. 4c, where each feature matrix $F_{i}^{s}$ is obtained by superimposing the input feature matrices that are expected to be adjacent, so that we have
  10 $$\begin{aligned} F_{i}^{s} = U\left( {W_{1}^{s}\bigotimes P_{i + 1}} \right) + W_{0}^{s}\bigotimes P_{i} + W_{- 1}^{s}\bigotimes P_{i - 1} \end{aligned}$$
  Where $D( \cdot )$ denotes the downsampling function. The convolution module of $W_{i}^{b}$ is the same as the top-down information path.
- Two-step fusion split information path The two-step fusion split information path is shown as Fig. 4d. Firstly, the higher and lower levels of the feature pyramid information are each combined into $\alpha _{s}$ and $\alpha _{t}$. We have
  11 $$\begin{aligned} \alpha _{s} = P_{4} + U\left( P_{5} \right) ,{~~~\alpha }_{t} = D\left( P_{2} \right) + P_{3} \end{aligned}$$
  After obtaining the combined features and fusing them by overlay stitching, we have
  12 $$\begin{aligned} \beta _{s} = W_{s}^{f}\bigotimes CON\left( {\alpha _{s},D\left( \alpha _{l} \right) } \right) ,~~\beta _{l} = W_{l}^{f}\bigotimes CON\left( {U\left( \alpha \right. }_{s} \right) \left. ,\alpha _{l} \right) \end{aligned}$$
  Where $W_{s}^{f}$ and $W_{l}^{f}$ is $3 \times 3$ conformable convolution module, and $CON( \cdot )$ denotes the stitching along the channel dimension. After these operations, the feature map $\beta _{s},\beta _{l}$ carries the information from the fusion of features at all levels. Finally adapting it to a multiscale pyramidal feature map, we have
  13 $$\begin{aligned} F_{2}^{f} = U\left( \beta _{l} \right) ,~~F_{3}^{f} = \beta _{l},~~F_{4}^{f} = \beta _{s},~~F_{5}^{f} = D\left( \beta _{s} \right) \end{aligned}$$
- Null information paths and cross-connected information paths Another characteristic of bone marrow oedema is its relatively concentrated location, so it is not necessary to use superimposed information paths in its detection to extract too much location information. Our algorithm adds 2 unparalleled information paths to reduce the complexity of the model. They are shown as Fig. 4e and f. The cross-connected path is used to perform direct mapping, while the null information path is used to eliminate redundant information paths. These two non-parametric information paths allow for a better balance between accuracy and efficiency.

#### Construction of the NAS-FPN model

**Fig. 5:**
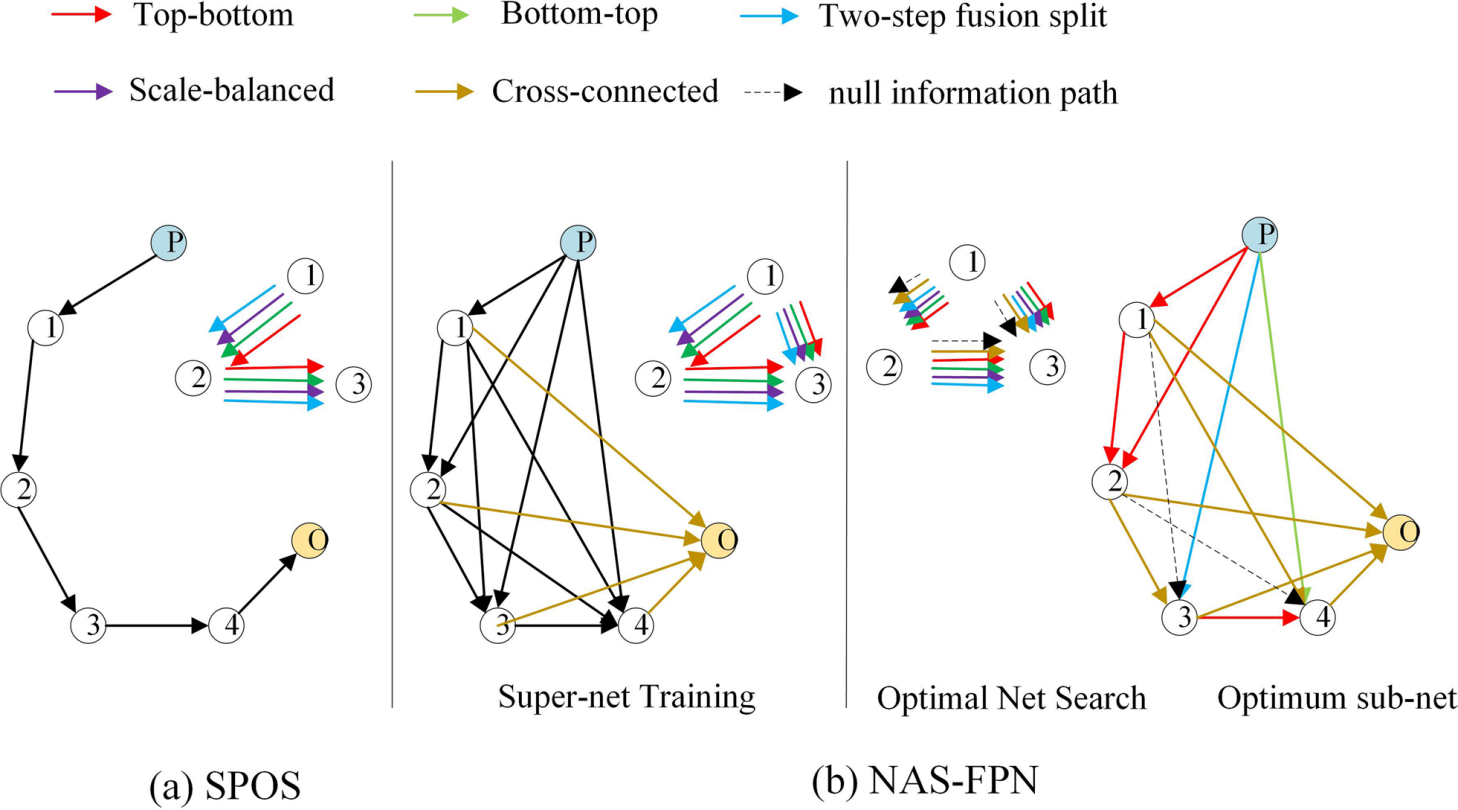
**a** FPN super-net structure for SPOS **b** NAS-FPN search space super-net results

In some recent studies, NAS has been applied to automatically search for FPN structures, the most common of which is the Single Path One-Shot [23]structure, as shown in Fig. 5a. However, there is only one information path between its nodes to connect and it has only sequential connections. From the information extraction point of view, an aggregated NAS can make more efficient use of the extracted information features.

The NAS-FPN is a fully connected DAG (directed acyclic graph). Each of its nodes represents an FPN with six different information paths connected between every two nodes. Where node *P* represents the feature matrix extracted from the backbone network and node *O* represents the final output feature matrix. In network training, our algorithm first constructs a super network containing all information paths, and then searches for the optimal sub-network through evolutionary algorithm. The optimal sub-network represents the optimal combination of FPN that aggregate multiple information paths. The optimal sub-network is searched by reasoning with previously trained weights rather than retraining the weights, so it is more efficient. In the experiments, we find that the model achieves the best results when $N=5$. When $N>5$, NAS-FPN can not increase the accuracy of the detection, but significantly decreases the speed of the detection. Therefore $N=5$ is used in the subsequent comparison experiments unless otherwise specified. In order to reduce the complexity of the network, we reduce the output channels of the FPN from 256 to 112. The corresponding channels of the network detection head are also reduced from 256 to 112. It is found experimentally that these channel reductions have less impact on the network accuracy, but can significantly reduce the weight size of the network (see the ablation data in the next section for details).

#### Optimization of super-net and loss functions of networks

The optimal subnetworks in Fig. 5b are searched by an evolutionary algorithm after completing the training of the super-net. That is, *S* subnets are first randomly selected from the super-net and the performance of each subnet is ranked by inferring the data from the validation set. Then the best *k* subnets start the “crossover” and “mutation” operations [24], and the new subnets are generated and the best subnet are selected after repeating the previous steps *n* times. The “crossover” refers to the crossover of two randomly selected subnetworks to generate a new subnet, and the “mutation” refers to the mutation of a random subnet boundary with probability *p* to generate a new subnet. In our algorithm, $S=50$, $k=10$ and $p=0.1$ are set. To help the convergence of the model, this algorithm adds an $L_1$ regularization function to the weights to balance the boundary losses. The total loss function is given by

14 $$\begin{aligned} L = L_{bbox} + \mu L_{1} = {\sum {\left( {L_{cls} + L_{loc}} \right) + \mu L_{1}}} \end{aligned}$$

Where $L_{cls}$ is the corresponding loss for lesion classification, which is the cross-entropy loss function; $L_{loc}$ is the corresponding positioning loss function, which is the Smooth $L_1$ loss function for $L_{loc}$; $\mu$ is the hyperparameter of the regularisation function $L_1$, and the network was found to work best when $\mu$ was taken as 0.11 in the experiments.

## Results and discussion

### Experimental data and experimental parameters

No publicly dedicated dataset is available for detecting bone marrow oedema in lumbar MRI images. The data for the research is obtained from the relevant department of Tianjin Hospital, and the lumbar MRI image dataset was obtained from 72 anonymous patients between 2019 and 2021. A total of 521 MRI images are screened, among which 416 images are placed in the training set and 105 images are placed in the testing set. The dataset was annotated according to the MS COCO [25] format, which was completed by three experienced radiologists. Our algorithm was trained using stochastic gradient descent. The RTX8000 GPU is used for accelerated training, and all experiments are carried out on this device. The training parameter batch-size is set as 4, the learning rate is set as 0.001, and a total of 30 training rounds were set. We used random flipping and random cropping as quality enhancement methods. Flip the mirror image of the original image randomly with a probability of 0.5. And clip the image randomly to 0.6–1.0 times of the original ones in size.

### Ablation experiments

**Table 1:** Effect of super net node *N* on precision and weight

Algorithms	Number of nodes *N*	*AP*_50_/%	Recall/%	F1/%	Weight size/MB	Single image detection time/ms
Faster-RCNN +FPN	–	84.9	91.7	88.2	322.54	161
Ours (Faster-RCNN + NAS-FPN)	2	83.7	91.2	87.3	265.66	135
	3	85.3	91.7	88.4	266.69	140
	4	87.3	91.2	89.2	270.65	140
	5	88.3	91.2	91.0	277.18	147
	6	88.0	91.3	90.8	304.19	158
	7	86.7	93.6	90.4	311.17	166

Ablation experiments are conducted to understand the contribution of each module in this paper’s algorithm. The ablation experiments use ${AP}_{50}$ of the COCO evaluation criteria as the evaluation metric. Besides, there are experimental data of recall and F1-measure as auxiliary verification data. Where ${AP}_{50}$ and recall are the average of five cross-validation experiments. The training set and verification set of each cross-validation are not repeated. The subjects of the ablation experiments include the number of super net nodes *N*, the deformable convolutional and deformable RoI layers and the NAS-FPN module.

The number of super net nodes *N* represents the number of levels of the FPN structure. On the one hand, its size directly affects the performance of the network. On the other hand, a larger *N* represents more network parameters, which affects the training speed and the weights. The experimental results are shown in Table 1. After adding the NAS-FPN module, the value of ${AP}_{50}$ increases 3.4$\%$ at most from 84.9$\%$. The recall of all experiments remained above 91.2$\%$. Meanwhile, F1-Measure has a maximum value of 91.0 when $N =5$. Also, the weight reduces 11.37 MB–56.88 MB. It proves the effectiveness of the NAS-FPN module. However, when $N \ge 5$, the ${AP}_{50}$ and the F1-Measure are not increasing, but the weights still increase rapidly and the detection rate decreases. To balance precision and efficiency, subsequent experiments use the value of $N=5$.

### Deformable convolution modules and NAS-FPN modules

**Table 2:** Enhancement of effects by deformable and NAS-FPN modules

Algorithms	Deformable Conv	NAS- FPN	*AP*_50_/%	Recall/%	F1/%	Weight size/MB	Single image detection time/ms
Faster- RCNN +FPN	–	–	84.9	91.7	88.2	322.54	161
Ours(Faster -RCNN + NAS-FPN)	$\surd$	–	85.7	90.4	88.0	327.10	140
	–	$\surd$	88.3	91.2	91.0	277.18	147
	$\surd$	$\surd$	90.6	95.1	92.8	275.21	144

In order to validate the improvement of the DCNAS structure on the detection of bone marrow oedema, we compare the performance of our algorithm with the Faster-RCNN+FPN. As shown in Table 2, when the deformable convolution module is added, ${AP}_{50}$ improves from 84.9 to 85.7$\%$, and the detection time is reduced to 140ms per image. When the NAS-FPN module is added, ${AP}_{50}$ improves to 88.3$\%$. When the DCNAS module is added, ${AP}_{50}$ improves to 90.6$\%$ and the detection time becomes 144ms per image, while the parameter weight is reduced from 322.54 MB to 275.21 MB compared to the unaltered network. When the deformable convolution and NAS-FPN are added, recall is also increased to 95.1$\%$, and F1-Measure is increased to 92.8$\%$. The experimental results show that the deformable module and NAS-FPN are effective.

### Algorithm comparison and analysis

**Fig. 6:**
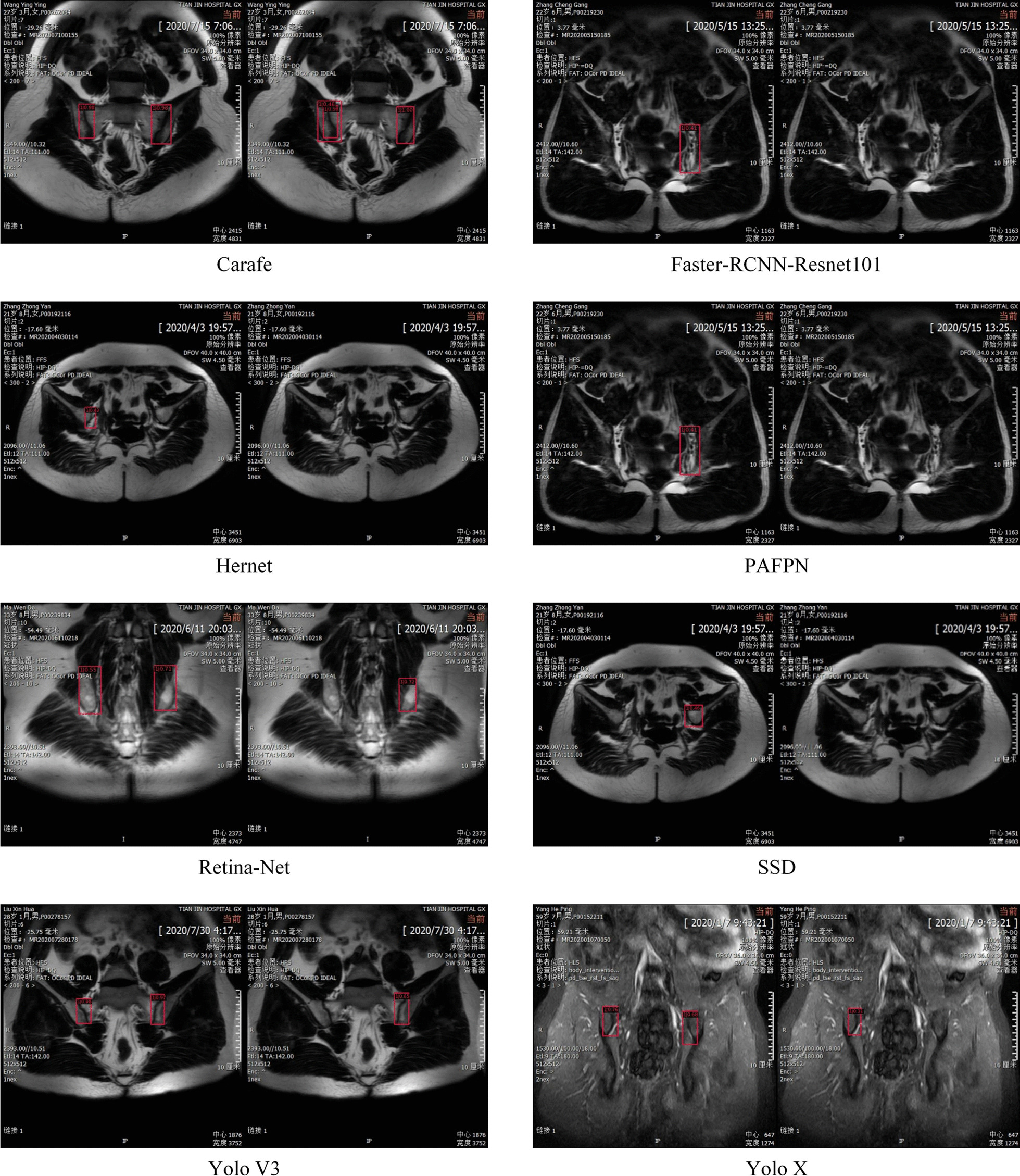
Comparison with other algorithms. The left side of each graph shows the results of our algorithm and the right side shows the results of other algorithms

In order to verify the effectiveness of the algorithm in this paper, the research performs comparative experiments with existing algorithmic models for target examination. Including classical networks such as Faster-RCNN-ResNet101, Retina-Net [26], SSD [27], Yolo V3 [28] and Yolo X [29]and some improved detection networks such as Carafe [30], Hernet [31], and PAFPN. The results are shown in Table 3 and Fig. 6.

**Table 3:** Comparison of current working algorithm with existing networks

Algorithms	*AP*_50_/%	Recall/%	F1/%	Weight size/MB	Single image detection time/ms
Carafe	83.3	88.4	85.8	366.34	144
Faster-RCNN-Resnet101	85.9	91.2	88.4	471.24	147
Hernet	88.0	93.4	85.8	367.41	178
PAFPN	83.8	88.9	86.3	350.21	140
Retina-Net	88.5	95.4	91.8	283.33	136
SSD	89.4	94.4	91.8	185.76	102
Yolo V3	87.0	90.9	88.9	481.40	109
Yolo X	86.9	93.4	90.1	105.30	109
Ours	90.6	95.1	92.8	275.21	144

As can be seen from Table 3, our algorithm has the best accuracy and F1-Measure for bone marrow oedema detection. and our algorithm also has good detection recall and speed. And our network weight is only 275.21 MB, which means it can be more easily deployed in the detection equipment of clinical medicine. Of course, it might be possible to use other lightweight network backbones with less weight. As can be seen from Fig. 6, our algorithm is able to detect bone marrow oedema in different styles of MRI images with few missed and false detections.

At present, the research on the detection of bone marrow edema in MRI images mainly focuses on CNN classification model. For example, Klontzas et al. [8] designed a CNN ensemble to detect bone marrow edema in bone joints. The accuracy of this ensemble for classification tasks is 82.18–93.68$\%$. Lee et al. [9] designed an optimization model based on ResNet18, which is used to detect bone marrow edema in sacroiliac joints. But this network also belongs to the category of image classification, and the detection accuracy of their model in the experiment is 93.55$\%$. Unlike their research, our work belongs to target detection. Our optimized neural network can not only classify abnormal MRI images accurately, but also give the location information of bone marrow edema as accurately as possible. This can better provide clinicians with reference information for auxiliary diagnosis. By now, there is almost no target detection model for bone marrow edema in MRI images. This is where our research leads. Compared with the classical target detection models, our model has achieved excellent results.

## Conclusion

Based on the Faster-RCNN, we redesigned part of the backbone network and the feature pyramid module. Extensive experiments have demonstrated that our algorithm can accurately detect bone marrow oedema in MRI images. At the same time, our algorithm balances accuracy and detection efficiency, and can also be relatively easy to deploy on hospital detection equipment. Therefore, our work has practical clinical implications and can be used as a diagnostic aid for doctors. In principle, our work can be used not only for the detection of bone marrow oedema, but also for the detection of other pathologies. In particular, the application of MRI images as a background for detection has good prospects. We will continue to design new dedicated networks and carry out new experiments in collaborating laboratories.

## Data Availability

The experimental data for this work has been presented in the paper. The dataset and algorithms have been deposited with the corresponding author; please contact the corresponding author for details if you wish to use them.

## Abbreviations

MRI: Magnetic resonance imaging
CAD: Computer aided detection
AUC: Area under curve
RoI: Region of interest
FPN: Feature pyramid networks
NAS: Neural architecture search
DAG: Directed acyclic graph
